# Performance of APSIM to Simulate the Dynamics of Winter Wheat Growth, Phenology, and Nitrogen Uptake from Early Growth Stages to Maturity in Northern Europe

**DOI:** 10.3390/plants12050986

**Published:** 2023-02-21

**Authors:** Uttam Kumar, Elly Møller Hansen, Ingrid Kaag Thomsen, Iris Vogeler

**Affiliations:** 1 Department of Agroecology, Aarhus University, 8830 Tjele, Denmark; 2 Grass Forage Science/Organic Agriculture, Institute of Crop Science and Plant Breeding, Christian Albrechts University, 24118 Kiel, Germany

**Keywords:** modelling, early growth stages, fertilizer management, parameters, variability, parameter sensitivity

## Abstract

Performance of the APSIM (Agricultural Production Systems sIMulator) wheat model was assessed to simulate winter wheat phenology, biomass, grain yield, and nitrogen (N) uptake for its potential to optimize fertilizer applications for optimal crop growth and minimal environmental degradation. The calibration and evaluation dataset had 144 and 72 different field growing conditions (location (~7) × year (~5) × sowing date (2) × N treatment (7–13)), respectively, and included seven cultivars. APSIM simulated phenological stages satisfactorily with both model calibration and evaluation data sets with r^2^ of 0.97 and RMSE of 3.98–4.15 BBCH (BASF, Bayer, Ciba-Geigy, and Hoechst) scale. Simulations for biomass accumulation and N uptake during early growth stages (BBCH 28–49) were also reasonable with r^2^ of 0.65 and RMSE of 1510 kg ha^−1^, and r^2^ of 0.64–0.66 and RMSE of 28–39 kg N ha^−1^, respectively, with a higher accuracy during booting (BBCH 45–47). Overestimation of N uptake during stem elongation (BBCH 32–39) was attributed to (1) high inter-annual variability in simulations, and (2) high sensitivity of parameters regulating N uptake from soil. Calibration accuracy of grain yield and grain N was higher than that of biomass and N uptake at the early growth stages. APSIM wheat model showed high potential for optimizing fertilizer management in winter wheat cultivation in Northern Europe.

## 1. Introduction

Winter wheat (*Triticum aestivum* L.) with its high yield potential and suitability to the environment is a widely grown cereal crop in most of northern and western Europe [1,2]. The development of high-yielding varieties, chemical fertilizers, pesticides, irrigation, and mechanization during the 1950s and 1960s has led to intensive farming systems with remarkably increased crop yields in many parts of the world [3]. While these intensive farming systems have resulted in higher economic returns, losses of nitrogen to aquatic environments have also increased in many areas, which remains a big concern worldwide [4]. The European Environment Agency reported [5] that between 2015 and 2017 groundwater nitrate concentrations in many European countries were higher than the maximum allowable limit of 50 mg NO_3_/L. Agriculturally intensive countries, such as Denmark, Germany, and Hungary, had higher nitrate concentrations than the countries with less intensive agriculture, such as Estonia, Norway, Finland, and Sweden. The primary cause of nitrate concentration in groundwater is nitrogen fertilizer inputs in the crop production systems [6,7].

To counteract groundwater contamination with nitrate, current agricultural practices need to be revisited to find a suitable balance between high yields and low environmental impacts. However, due to the complexity of several interacting factors (e.g., temporal N supply of the soil, plant uptake, soil moisture and temperature, and their interactions) identifying sustainable solutions and recommendations is not easy [8]. Because of that, optimal site-specific information on the nitrogen requirements for high crop production with minimal N losses to the aquatic environment is lacking. To evaluate and understand the effect of various mitigation options, one approach is to conduct long-term field experiments on a large scale [9]. In the context of economic feasibility and practicality, such experiments are difficult to execute on a large scale [10]. Alternatively, using advanced tools, such as crop simulation models, can reduce the usage of resources and time and still deliver similar valuable information [11].

Deterministic crop simulation models (hereafter crop models) of the soil–plant–atmosphere continuum provide an advanced and holistic way to study the effect of N fertilizer management practices on various crops and soil processes. Due to a process-based foundation, crop models have been widely used to address research questions in the areas of climate change and variability on crops [12], soil organic carbon [13], optimizing farming practices [14], crop–livestock systems [15], decision making and farmer advisory [16], plant nitrogen status [17], and for guiding N fertilisation based on soil N amounts or pasture N concentrations to reduce N leaching [18,19]. However, calibrating a crop model to address a research question is a complex and challenging task. Different crop models use different approaches to formalize algorithms to describe processes which, evidently, make them produce different results, even when supplied with the same set of input data. Besides differences in the algorithm formalisms, parameter values in the models are not universally applicable [20]. Calibrating key parameters, therefore, is a critical step to ensure the robustness of a model when used for scenarios other than those tested [21,22].

The APSIM model (Agricultural Production Systems sIMulator) is a process-based crop model and has been widely used globally for various aspects of crop production and decision making [23,24]. Recently, Vogeler et al. [25] used APSIM to test its performance in capturing the effects of variable sowing dates on N leaching for general winter cereals (i.e., winter rye and winter wheat) in Denmark and Germany. In this study, simulations for biomass, grain yield, grain N, and cumulative N leaching were assessed by using default values for most parameters, while parameters related to phenology (thermal time requirements between phenological stages), N concentration in leaf and stem, and grain yield (grains_per_ gram_stem) were adjusted to reflect values in the observed data. The model performed fairly well for simulating biomass, grain yield, grain N at harvest, and cumulative N leaching over several years, but not for individual years. Calibrating crop models, including data from early growth stages until harvest, to better capture the dynamics of biomass accumulation and N uptake during crop development could improve the prediction robustness for individual years.

A potential option for reducing N losses is by better matching the dynamics of plant growth and N requirements with N availability in the soil and fertilization rates. This requires data on plant growth, N uptake, and soil N at regular intervals. Since the common fertilization practice is to apply N during the early growth stages in spring, models need to predict biomass and N uptake adequately during these stages when used for temporal and site-specific N recommendations. APSIM has not been rigorously tested for simulating winter wheat under Northern European conditions, especially for the early growth stages. Thus, calibration of parameters that influence phenology, biomass accumulation, grain yield, and N dynamics throughout the season is needed. Therefore, this study was conducted to calibrate and evaluate performance of the APSIM winter-wheat model using data from a wide range of N fertilization rates and sites with different climatic conditions in Denmark to assess its potential for devising fertilizer management strategies for optimal crop growth and minimal environmental degradation in future.

## 2. Results

### 2.1. Calibration and Evaluation of Phenology

**Figure 1:**
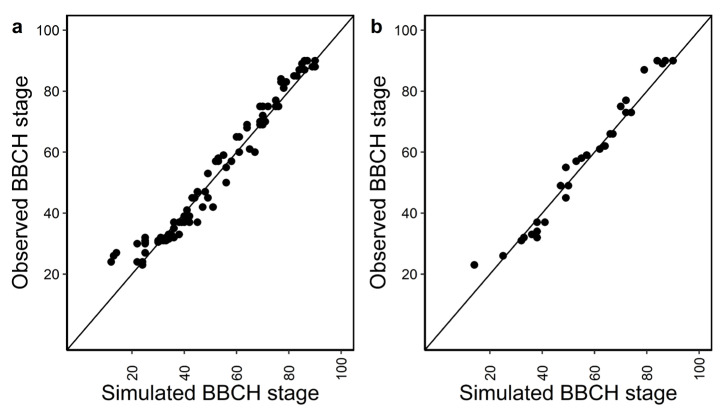
Performance of APSIM winter wheat with current set of calibrated parameters to simulate phenology with (**a**) calibration and (**b**) evaluation data sets using cultivar Dan_winter. Note: BBCH stages from the simulations were extracted based on the date of observations in the field.

APSIM simulated phenology development from as early as BBCH 23 (three tillers visible) to physiological maturity, BBCH 90, very satisfactory with r^2^ and NSE close to one, at seven locations, for five years, and two sowing dates under calibration and evaluation (Figure 1a,b and Table 1). There were slight underestimations for very early BBCH stages in both calibration and evaluation data sets. In about 84% of cases, the default cultivar reached BBCH 90 on average 9 days earlier than the observed dates. Simulations with the modified cultivar had similar maturity dates as in the calibration data set, but on average matured 5 days later compared to the maturity in the validation data set. Overall, phenology simulations were better in terms of r^2^, NSE, and RMSE with the calibrated set of parameters for Dan_winter in this study than the parameters set for default Batten_winter and modified Batten_winter in Vogeler et al. [25] (Table 1).

### 2.2. Calibration and Evaluation of Biomass and N Uptake

**Figure 2:**
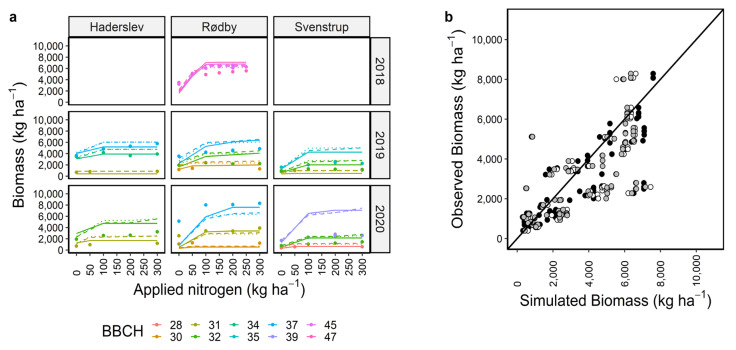
Calibration of the APSIM winter wheat biomass under 7–13 nitrogen management scenarios at different BBCH stages under three locations and years. Dots are observed data points and solid, dashed, and dotted lines are model outputs with the current calibrated set of parameters for Dan_winter, default, and modified cultivars, respectively (**a**). Black, white, and grey circles are for Dan_winter, default, and modified cultivars, respectively (**b**). Diagonal black line is 1:1 line (**b**). Discontinuous lines at 200 N application indicate the simulation outputs of single dose or split applications at the locations (Table S1). For Rødby, discontinuous line also indicated two simulations with different sowing dates.

**Figure 3:**
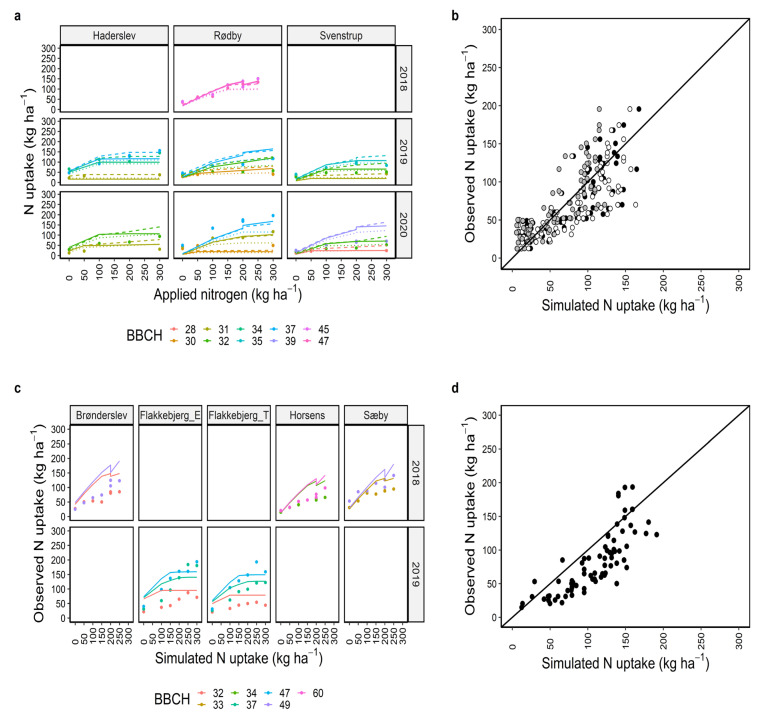
Calibration (**a**,**b**) and evaluation (**c**,**d**) of APSIM winter wheat model for N uptake under 7–13 nitrogen management scenarios. Coloured circles are observed data points and solid, dashed, dotted lines are model outputs with the current set of calibrated parameters for Dan_winter, default, and modified cultivars (**a**,**c**). Black, white, and grey circles represent model outputs using Dan_winter, default, and modified cultivars, respectively (**b**,**d**). Diagonal black lines in b and d are 1:1 lines. Discontinuous lines at 200 kg N application indicate the simulations output with single dose or split applications of N at the locations in the same year (Table S1). For Rødby in 2018, discontinuous line also indicated two simulations with different sowing dates.

The simulation performance of APSIM with the Dan_winter cultivar for biomass across locations, years, and nitrogen management scenarios under calibration was satisfactory with r^2^ of 0.66 and RMSE of 1510 kg ha^−1^ (48% of the observed mean) (Figure 2b, Table 1). When inspecting the performance for individual locations and years, mixed responses can be seen for different stages, with no particular pattern. For example, parameters for the Dan_winter cultivar underestimated biomass at BBCH 37 for Rødby in 2020 and slightly overestimated it in 2019 (Figure 2a). For BBCH 32, biomass simulations were close to the observed data for Svenstrup in 2019 but it was overestimated for Haderslev in 2020. Similarly, APSIM with the Dan_winter cultivar simulated N uptake satisfactorily both for calibration and evaluation data sets (Figure 3b,d, Table 1) with r^2^ of 0.64–0.67 and RMSE 28–39 kg ha^−1^ (~39–54% of the observed mean). Simulated N uptake at different BBCH for individual locations and years were similar with different fertilizer applications (Figure 3a) as for biomass under calibration (Figure 2a). There was a tendency to overestimate N uptake with higher N applications under validation (Figure 3c,d). Overall, simulations with the Dan_winter cultivar were not very different than the simulations with the default and modified cultivars (Table 1). However, in simulations with modified cultivars, there were more cases of underestimation of N uptake (Figure 3b).

### 2.3. Calibration and Evaluation of Grain Yield and Grain Nitrogen

**Figure 4:**
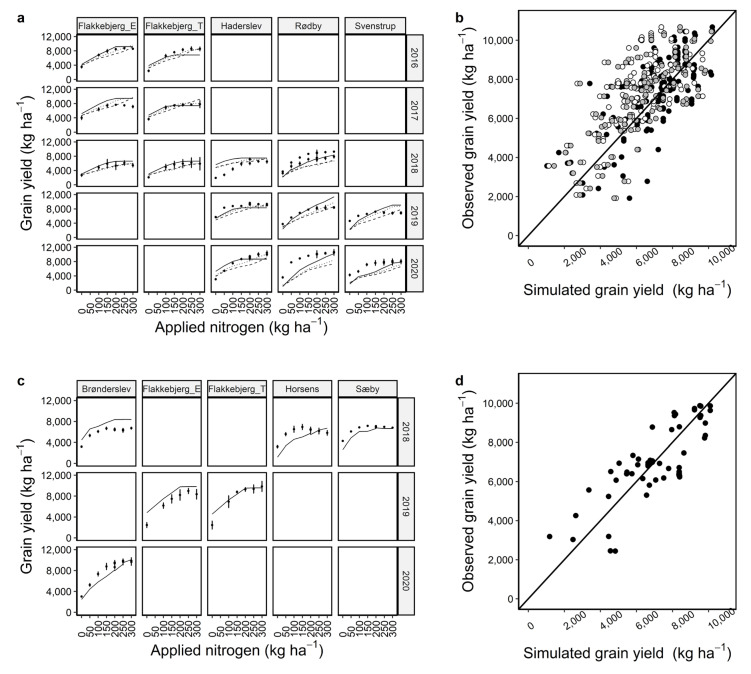
Calibration (**a**,**b**) and evaluation (**c**,**d**) of APSIM winter wheat grain yield under 7–13 nitrogen management scenarios. Coloured circles are observed data points and solid, dashed, and dotted lines are model outputs with Dan_winter, default, and modified cultivars, respectively (**a**,**c**). Black, white, and grey circles represent model outputs using Dan_winter, default, and modified cultivars, respectively (**b**,**d**). Diagonal black lines in b and d are 1:1 lines. Discontinuous lines (e.g., Brønderslev and Sæby) at > 200 kg N application indicate the simulations output with single dose or split applications of N at the locations in the same year (Table S1). For Rødby in 2018, discontinuous line also indicated two simulations with different sowing dates.

**Figure 5:**
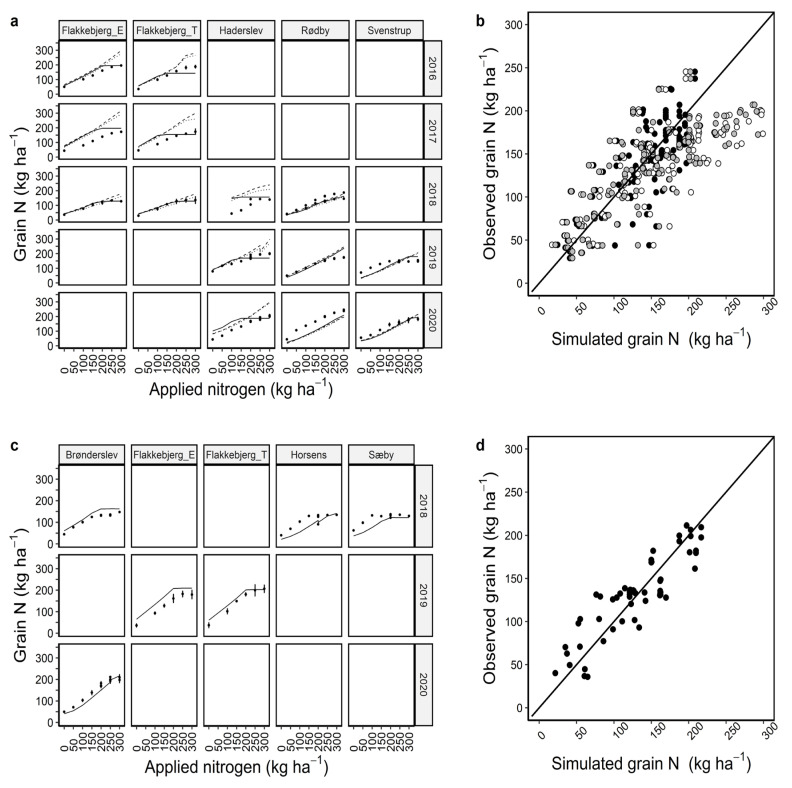
Calibration (**a**,**b**) and evaluation (**c**,**d**) of APSIM winter wheat grain N under 7–13 nitrogen management scenarios. Black circles are observed data points and solid, dashed, and dotted lines are model outputs with Dan_winter, default, and cultivars, respectively (**a**,**c**). Black, white, and grey circles represent model outputs using Dan_winter, default, and modified cultivars, respectively (**b**,**d**). Diagonal black lines in b and d are 1:1 lines. Discontinuous lines (e.g., Brønderslev and Sæby) at >200 kg N application indicate the simulation outputs with single dose or split applications of N at the locations in the same year (Table S1). For Rødby in 2018, discontinuous line also indicated two simulations with different sowing dates.

The current set of calibrated parameters simulated grain yield and grain N better than the biomass and N uptake both under calibration and evaluation considering RMSE, r^2^, and NSE together. For grain yield, r^2^ was 0.51 and 0.61, and RMSE was 1491 and 1296 kg ha^−1^ (18% of the observed mean) (Figure 4b,d) and for grain N, r^2^ was 0.60 and 0.68, and RMSE 32 and 25 kg ha^−1^ (18–23% of the observed mean) (Figure 5b,d) across the locations, years, and N management scenarios under calibration and evaluation, respectively (Table 1). The grain yield and grain N simulations for individual locations and years under calibration showed that the simulations were mostly in line with the observed data under most of the N treatments (Figure 4a and Figure 5a). However, there were a few under (e.g., for Flakkebjerg_T in 2016 and Rødby in 2020) and overestimations (e.g., Rødby in 2019, Flakkebjerg_E in 2017, Bronderslev in 2018, Figure 4a,c). Grain N was underestimated for Horsens and Sæby in 2018 and Brønderslev in 2020 with the evaluation data set (Figure 5c). The r^2^ and RMSE under evaluation were better than under calibration (Figure 5d, Table 1).

Simulations using default and modified cultivars usually had more cases of underestimations of grain yield than those using the Dan_winter cultivar (Figure 4a,b). Additionally, simulations with those cultivars overestimated grain N for several cases where observed grain N was between 150–200 kg N ha^−1^ (Figure 5a,b). Overall, model evaluation statistics showed that simulations with the Dan_winter cultivar were better than default and modified cultivars (Table 1).

### 2.4. Inter- and Intra-Annual Variability in Observed and Simulated Data

The comparison of inter- and intra-annual variability (standard deviation, SD) between simulation and observed data was conducted to find the reasons for mixed responses for both biomass and N uptake during growth stages between BBCH 31 and BBCH 45 in the model. Variability across locations, years, BBCH stages, and fertilizer treatments in the simulation data set was higher than in the observed data set (Table 2). Further investigation of the inter-annual variability for two locations, Rødby (from BBCH 28–47) and Svenstrup (from BBCH 28–39), across all fertilizer treatments showed that simulation variability for biomass was still higher. We further computed inter-annual variability from two years (2019–2020) of simulations for one BBCH stage and one fertilizer treatment (300 kg ha^−1^) and indicated a higher variability if the difference in SD between simulation and observed SD were higher than 20% of the observed SD. Inter-annual variability was 263% higher than the observed variability for BBCH 31 for Haderslev, and it was 49% higher for BBCH 32 for Svenstrup. Similarly, intra-annual variability for two BBCH stages in the same season (2020) showed that the variability in simulations was 10% higher for Rødby (BBCH 31 and 37) and 26% higher for Haderselv (year, 2019, BBCH 34 and 37). Similar to the variability for biomass in simulations, the variability for N uptake was also higher in all variability testing criteria.

### 2.5. Sensitivity Analysis to Assess Overestimation of N Uptake

**Figure 6:**
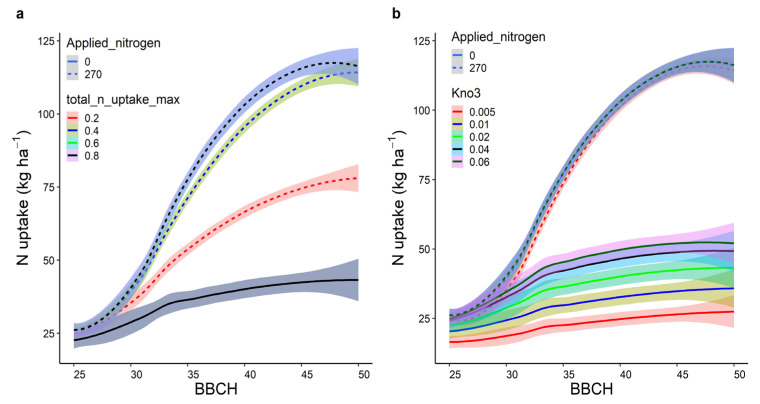
Sensitivity analysis of total_n_uptake_max (**a**) and kno3 (**b**) (g m^−2^ d^−1^) regulates maximum total N uptake based on root depth, and soil layers and their depths, respectively. Solid and dashed lines represent mean of three years (2016–2018) and shaded area is standard error. Applied nitrogen was in kg ha^−1^.

Sensitivity analysis on the parameters that regulate critical and upper bounds of N concentration in leaf (n_conc_crit_leaf and n_conc_max_leaf) and stem (n_conc_crit_stem and n_conc_max_stem) showed that these parameters are stable and have little influence on the variations for N uptake during early growth stages (Figures S1 and S2). However, the sensitivity analysis of total_n_uptake_max and kno3 indicated that these parameters are highly sensitive for N uptake (Figure 6a,b). When total_n_uptake_max value was set to the default of 0.6 or higher, the sensitivity was low. In contrast, values < 0.6 showed large differences in N uptake throughout the plant development with a higher amount of applied nitrogen. There was no variation in N uptake with zero fertilizer application. In contrast, the sensitivity analysis of *kno3* showed an opposite response. It was more sensitive to zero N application than to 270 kg N ha^−1^.

## 3. Discussion

### 3.1. Performance of APSIM for Simulating Phenology and Early Stage Biomass and N Uptake

Calibration with less detailed and smaller data sets affect prediction accuracy of crop models [26,27]. Data to calibrate dynamics of winter wheat phenology, N uptake, and growth and productivity variables in Northern Europe from early growth stages to maturity are scarce. This has limited robust calibration and evaluation of crop models and their applications in identifying fertilizer management strategies for reducing N inputs and environmental degradation such as groundwater contamination. In this study, data on winter wheat phenology, biomass accumulation, N uptake, grain yield, and grain N from early growth stages to maturity were obtained from two sources: (1) field trials conducted in farmers’ fields and (2) research stations, and the APSIM wheat model was calibrated and evaluated using the data for its potential to be used for fertilizer management strategies for reducing nitrate leaching in future.

Winter wheat is sown in autumn, around September, and fertilizers are applied during the following spring months in Northern Europe. Therefore, crop models need to predict early growth stages, biomass accumulation, and N uptake in spring adequately to be robust and reliable for their applications. The APSIM wheat model predicted phenology accurately with only a few cases of underestimations during early BBCH stages for both calibration and evaluation data sets. The trials were conducted on farmers’ fields to observe grain yield and N uptake to find the balance between optimal crop growth, fertilizer application, and environmental degradation. Due to this, the exact measurements of physiological maturity were not recorded. The same approach was adopted for the trials in the experimental stations at Flakkebjerg and the same variables were measured. Thus, for the model calibration, physiological maturity was set to the date of harvest (recorded as BBCH 90). This might have created an unquantifiable and unverifiable degree of uncertainty, which could be the reason for the slight underestimation of terminal stages. When simulations with the calibrated parameters for the Dan_winter cultivar were compared with the default cultivar, Batten_winter, and the modified Batten_winter cultivar used in Vogeler et al. [25], we found that Dan_winter gave similar or better phenology outputs. It can be argued that due to discrepancies in BBCH 90 measurements such a conclusion cannot be formed. In addition to the better performance of Dan_winter under validation data set for phenology, the simulations were also better for the grain yield and grain N than the simulations with default and modified cultivars. The underestimations of the grain yield with default and modified cultivars could be linked with early maturity (on average 5–9 days) than the observed maturity days, thus, lower grain yields. Therefore, it can be concluded that the current set of calibrated phenology parameters is more accurate.

The performance of APSIM regarding phenology was particularly accurate and robust for BBCH 31 and 37 which are usually the target growth stages for fertilizer application in spring [25,28]. Furthermore, one set of calibrated parameters accurately simulated the dynamics of phenology from early to physiological maturity for seven different cultivars that were grown at seven locations in Denmark. This indicates that these parameters can be used for winter wheat cultivars with similar phenology across Northern Europe for a wider application of the model.

In traditional agronomy, information on biological optimal crop N uptake has been used for computing critical N demand and designing crop N management for obtaining optimal biomass [29]. Crop models have also been used to assess plant nitrogen status and guide N fertilization to reduce N leaching [17,18]. In this study, the APSIM simulated biomass and N uptake during the early stages of spring reasonably well. However, better accuracy for biomass and N uptake simulations for growth stages around tillering (BBCH 28–31) and booting (BBCH 45–47) was observed than in between growth stages (BBCH 32–39). The good accuracy at booting, when plants experience a sudden increase in N demand due to the subsequent development of grains, is reassuring that APSIM can be used to refine N fertilisation rates during early growth stages. Simulation accuracy of biomass and N uptake followed a similar pattern with growth stages, which shows the interdependence of biomass and N and is not surprising as APSIM, like other crop models (e.g., DSSAT, [30]), allocates N based on dry matter accumulation and growth stage [31,32]. There were not enough data to evaluate biomass simulations. Nevertheless, a similar pattern of biomass and N uptake simulations under calibration and the dependency of N concentration on biomass indicates that the calibration accuracy for biomass prediction is likely to be robust under wider growing conditions.

The higher inter- and intra-annual variability in the simulation outputs than the observed data for biomass and N uptake during early growth stages suggest that responses for biomass and N uptake could be related to different metabolic and structural demands of N in different organs generated by interactions ofclimatic and soil characteristics in different years [33] and was probably not well captured in the model. High inter-annual variability for N uptake in most cases (e.g., 400–550%) can also explain the tendency to overestimate N uptake in the evaluation data set.

The difficulty in accurately measuring biomass at early stages adds another level of uncertainty. Due to the extreme variability of individual plants even at a small scale, the determination of grass biomass is very difficult [34], and this could also be expected at early growth stages for winter wheat in Northern Europe. APSIM also does not account for the increasing ratio of diffused light with increasing latitude for biomass production, which can affect the balance between photosynthesis and respiration [24]. Therefore, mixed responses for the simulations of biomass, overestimation for N uptake, and high inter-annual variability could also be associated with the uncertainty in the observed data and model formalism to simulate biomass accumulation.

Most formalisms of algorithms in crop models are typically based on the declining concentration of N in dry matter with crop development (e.g., [35]). Such functions are usually defined by upper and lower bounds, which may have different empirical robustness during crop development [32]. An earlier study by Hansen et al. [36] showed that bound defined functions might underestimate the N concentration, especially in early growth stages. In contrast, Justes et al. [29] reported that bound-based N uptake is better in capturing the variability of the observed N in the crop based on dry matter. Overall, r^2^ of 0.64–0.66 indicate that the bound-based approach in APSIM captured the N uptake relationship between simulations and observations accurately and in line with the findings of Justes et al. [29]. In the calibration process with the modified cultivar in Vogeler et al. [25], parameters that regulate lower, upper, and middle bounds of N concentration in the leaf and stem were adjusted, which might be the reason for lower RMSE for N uptake compared with calibrated parameters for Dan_winter (Table 1).

The APSIM wheat model under non-limiting conditions can access all soil nitrogen within the root zone (by default only the nitrate form of nitrogen is taken up) regulated by the parameter total_n_uptake_max. Under limiting N conditions, the extracted N from individual soil layers is regulated by parameter kno3. However, in reality, plants cannot access all nitrate available in the soil at early growth stages due to a less developed root zone [37]. Such implementation in the model likely overestimated N uptake during early stages, particularly with the higher fertilizer inputs. However, the overestimation of N uptake during these stages was not transferred or reflected in grain N (discussed in the following section). When total_n_uptake_max value was set to the default of 0.6 or higher, the N demand by the crop was satisfied, hence, the sensitivity was low. In contrast, values < 0.6 showed large differences in N uptake throughout the plant development with a higher amount of applied nitrogen. There was no variation in N uptake with zero fertilizer application as without N fertilization soil mineral N would be limiting the uptake regardless of differential access to N from the root zone. In contrast, *kno3* was more sensitive to zero N application than to 270 kg N ha^−1^, which suggested that with sufficient N variable access to N from the root zone does not affect N uptake much. However, under limited N conditions differential access to N from the soil, based on different values of *kno3*, the plant produces variations in N uptake. The sensitivity analyses suggest that the overestimation of N uptake during early growth stages may be reduced or eliminated if the parameters *total_n_uptake_max* and *kno3* are included in the calibration process. However, caution to calibrate crop-specific parameters such as *total_n_uptake_max* is always recommended [38,39]. We computed a maximum N uptake across fertilizer applications, sites, and years during early stages (BBCH 28–49) from the observed data based on 20 g m^−2^ on 235 days after sowing. The value was 0.09 g m^−2^ d^−1^. This value was much lower than the default value of 0.6. Considering satisfactory simulations for grain yield and grain N with default values of *total_n_uptake_max* and *kno3*, we did not calibrate these parameters.

### 3.2. Grain Yield and Grain N Simulation Capacity of APSIM

In this study, similar or better r^2^ and RMSE values for grain yield and grain N under the evaluation data set compared with the calibration data set, and better performance than the default and modified cultivars, indicate that the prediction accuracy of calibrated parameters was robust for Danish conditions and that the calibrated model can be used for a wider range of scenarios across Northern Europe. Better performance for grain yield and grain N simulations than for biomass and N uptake (based on lower RMSE) for early growth stages can be explained by the fact that the development of equations/parameters for predicting grain yield and grain N in the models were performed by utilizing, usually, larger data sets in different growing conditions compared to the equations/parameters that were developed to predict biomass and N uptake in the earlier growth stages, as well as the above mentioned difficulty in accurately determining biomass during early growth stages. Model calibration with a smaller data set can lead to a high degree of prediction errors [21]. A larger available data set for grain yield and grain N calibration than biomass and N uptake might be another reason for robust simulations of grain yield and grain N [26].

Grain N compared with N uptake during early growth stages is regulated by more numbers of parameters related to grain development, including grain number, grain size, thermal time for grain filling, and potential grain filling rate. Besides the interdependency of these grain development parameters, they are also linked to N availability in the soil. Thus, the role of a larger data set, the parameters, and the formalism of the algorithm for grain N prediction appear to contribute to the prediction robustness more than the parameters and formalism of the mechanism that predicts N uptake in the early growth stages.

The overestimation of N uptake during early stages was not translated to grain N. This further suggested the differences in the parameters and their sensitivities to environmental factors (e.g., temperature) for simulating grain N during grain development and N uptake during the vegetative phase. As mentioned above, grain yield and grain N calibration were performed in the third step after calibrating phenology, biomass, and N uptake. Calibration to achieve better performance of grain N resulted in a loss of accuracy for grain yield and N uptake, a problem generally associated with multi-objective calibration [40].

## 4. Materials and Methods

### 4.1. Description of Field Trials

**Figure 7:**
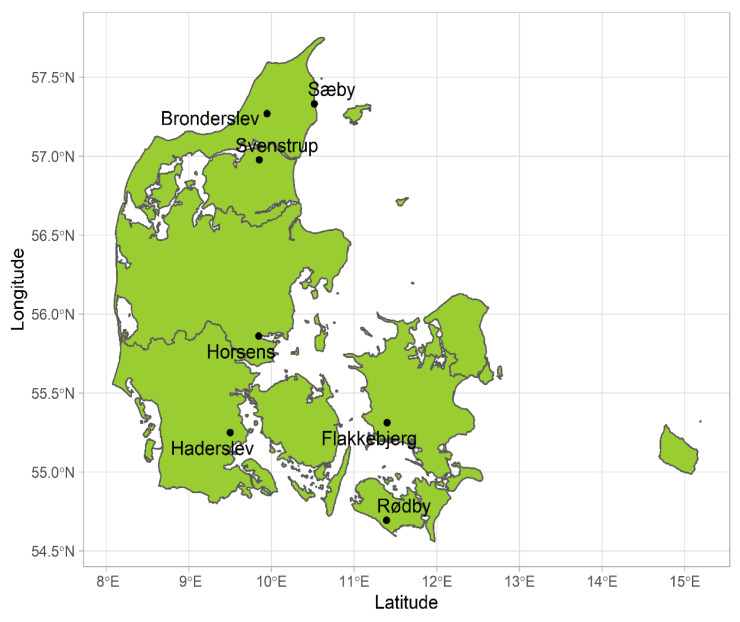
Locations of the field trials for data collection for model calibration and evaluation.

The winter wheat trials were conducted at seven locations: in Rødby and Haderslev from 2018 to 2020, in Svenstrup from 2019 to 2020, in Flakkebjerg from 2016 to 2019, in Brønderslev for 2018 and 2020, and in Sæby and Horsens for 2018 (Figure 7). In Flakkebjerg, the trials were conducted at the Aarhus University research station and repeated each year in the same fields. At the other sites, the trials were set up on farmers’ fields (conducted by SEGES innovation (https://en.seges.dk/About-us, accessed on 21 April 2021), and different nearby fields were used each year. These trials were conducted under 7–13 different N treatments to investigate the effect of N rate on yield and N uptake. N application was applied in two broad approaches, with either single-dose application, or split applications with either 2 or 3 application timings. Each N treatment was replicated 3–4 times. Locations, sowing dates, wheat cultivars, and N treatments in the trials used for model calibration and evaluation are presented in Table S1 (Calibration) and Table S2 (Evaluation), respectively. In Flakkebjerg, crops were either sown on time (Flakkebjerg_T) according to common practice at the end of September, or three weeks earlier (Flakkebjerg_E). The crop was only sown on time at the other sites. The cultivars used in the study were all of the same maturity class (https://www.landbrugsinfo.dk/public/c/9/5/planter_afgroder_sortinfo_dk, accessed on 29 August 2022). However, they differed slightly in yield with differences in grain yield within −9% to +7%, and in dry matter crude protein within −7% to +8% relative to the mean of reference cultivars in the national trials. To control pests, diseases, and weeds recommended amounts of insecticides and herbicides were applied.

### 4.2. Data Collection

Phenological development was assessed based on observations of events or growth stages following the BBCH scale (BBCH scale was developed jointly by BASF, Bayer, Ciba-Geigy, and Hoechst for numeric coding of growth stages that can be readily adapted to all crops, [41]). Depending on the location and years 5–11, growth stages were visually assessed during the growth cycle of the crop (Table 3) and corresponding dates were recorded. Sampling occasions for the measurements of biomass, N uptake, grain yield, and grain N are provided in Table 3. Biomass and N uptake measurements in spring were performed on plant samples cut approximately 1 cm above the soil surface from an area of 0.25 m^2^. Eight samples were taken from each treatment and these samples were pooled before N analysis from farmers’ fields, except at Flakkebjerg where samples from each plot from four replications were analysed separately for N concentration. All plant samples were dried at 60 °C before analysis. Grain yield and grain N measurements were performed from the samples harvested from an area of at least 15 m^2^ in each plot. Samples from Flakkebjerg were analysed for total N (Dumas) on a flash 2000 organic elemental analyzer in the laboratory at the Aarhus University campus Viborg. Near-infrared transmission (NIT) was used to estimate protein content in the samples from farmers’ fields [42]. The N content in grains was afterward calculated using a protein factor of 5.70 for wheat [43].

### 4.3. Soil Characteristics

Data on soil texture, pH, wilting point, field capacity, saturation point, and organic matter were obtained from two studies [44,45]. An overview of the extracted soil characteristics from the above sources and initial available water and nitrogen content for the locations used in APSIM is presented in Table 4. The soils at most locations are sandy or sandy loams (51 to 77% sand in topsoil), with the highest silt content at Sæby and the highest clay content at Haderslev.

### 4.4. Weather Data

**Figure 8:**
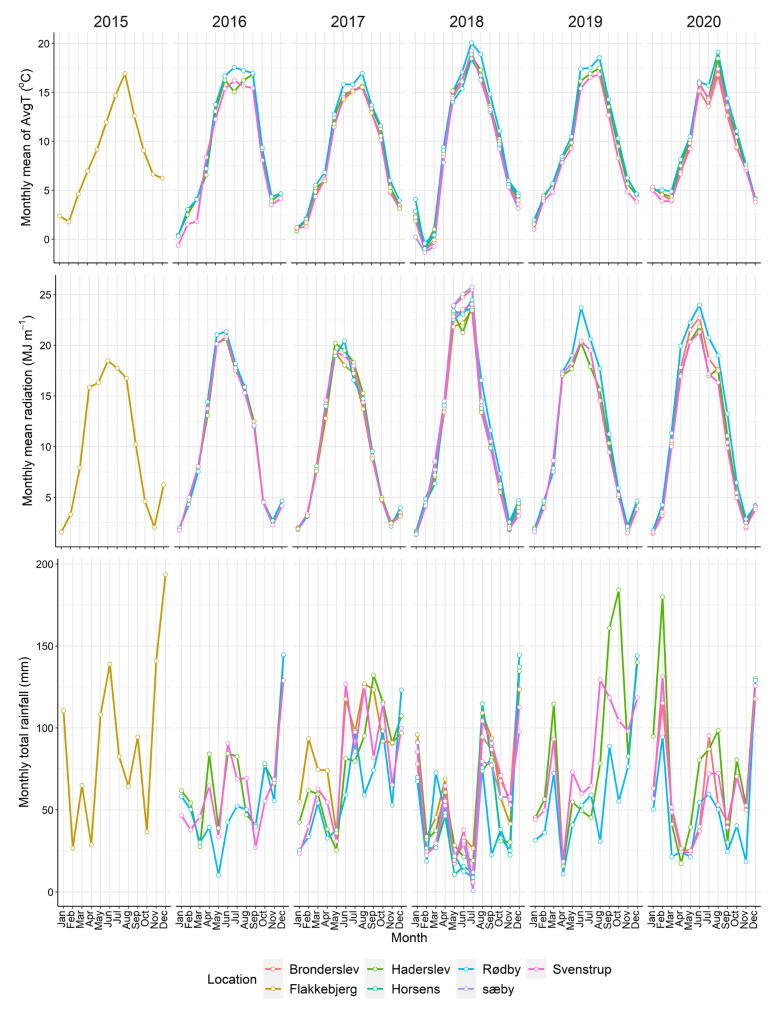
Monthly mean of average temperature (AvgT), radiation, and rainfall of calibration and evaluation locations for the experimental years at each location from 2015 to 2020 are presented in the upper panel, middle, and lower panel, respectively.

Weather data were collated from two sources from 2015 to 2020. Data on global radiation and rainfall were obtained from the online gridded (10 × 10 km) database (http://agro-web01t.uni.au.dk/KlimaDB/, accessed on 21 November 2021) managed by Aarhus University. Data on maximum and minimum temperature were obtained from the website of the Danish Meteorological Institute for the nearest weather stations to the studied locations. The weather stations were Lolland (for Rødby), Haderslev (for Haderslev), Aalborg (for Svenstrup), Tylstrup (for Brønderslev), Stenhøj (for Sæby), and Horsens (for Horsens). The monthly average temperature and radiation showed little differences among the locations (Figure 8). Rødby had the highest average temperature and radiation from June to August in some years compared with the other six locations. The differences in rainfall were more visible among the locations, however, without any particular trend. In contrast to temperature and radiation, Rødby had the lowest rainfall in most of the years.

### 4.5. APSIM Model Description

APSIM (classic version, APSIM 7.10) is a process-based deterministic crop model that simulates crop growth and development, and carbon and nitrogen dynamics in the soil, and plants at a daily time step depending on climatic conditions and agronomic inputs [46]. APSIM crop model (in this case wheat model) primarily simulates crop development using a thermal time approach to identify different phenological stages and phases. Vernalisation and photoperiod factors are incorporated into phenology algorithm to account for their respective effects during vernalisation and photoperiod sensitive stages. Water and nitrogen stresses affect leaf appearance rate (phyllochron), which, depending on intensity, can delay phenology during the vegetative stages.

Biomass assimilation is calculated from intercepted radiation by leaf area which is multiplied by radiation use efficiency factor [47]. To account for temperature, water and N stress biomass reduction factors are incorporated into the algorithm. After computing the available biomass for growth, it is then partitioned into leaf, stem, roots, and pods (grain) based on growth stage and partitioning factors. Depending on the available biomass for grain development, grain yield is simulated by interacting parameters regulating kernel number, grain growth, and grain filling rate. N concentration (demand) in different plant organs is primarily simulated based on the dry weight of the organ during the early growth stage, while after anthesis, N concentration in the grain is simulated by the parameter potential_grain_n_filling_rate together with the parameters that regulate grain development. The APSIM crop model interacts with SurfaceOM (surface organic matter), SoilN (soil nitrogen), SoilWat (soil water) models to simulate the dynamics of N, C, and water, respectively, with manager scripts accounting for fertilizer, manure, and crop residue input [48]. More description of the wheat model can be found online (https://www.apsim.info/wp-content/uploads/2019/09/WheatDocumentation.pdf, accessed on 31 July 2022).

### 4.6. APSIM Winter Wheat Model Calibration and Evaluation

A combination of sowing dates, N treatments, locations, and years created 144 growing conditions for calibration (Table S1) and 72 growing conditions for evaluation data sets (Table S1). A larger data set was used for calibration in order to capture broader variabilities in the response variables. This approach improves the prediction robustness and makes the model applicable to wider scenarios [26]. An overview of the date of measurements on biomass, N uptake, grain yield, and grain N under each N treatment during the crop cycle is provided in Table 3. There are several default spring and only a few winter wheat cultivars in APSIM but none of them represent a cultivar that is grown in Danish conditions and was calibrated for phenology, biomass, grain yield grain nitrogen, and nitrogen uptake in a detailed way. Initially, we tested a default cultivar Batten_winter in APSIM that represents winter wheat and a cultivar (modified Batten_winter) created in a recent study conducted in Denmark [25]. Although modified Batten_winter performed better than the default Batten_winter, both cultivars did not simulate the variables of interest satisfactorily. Therefore, we created a new cultivar using the parameters based on three criteria: (1) expert knowledge and information on the parameter, (2) their direct influence on measured variables, (3) frequent use in earlier studies (e.g., [47,49,50]). Such an approach to select the parameters for calibration is common [27]. Estimation of parameter values was performed manually within a reasonable range by changing one parameter at a time with a trial-and-error approach similar to earlier studies [51,52]. Following the most common calibration approach [27], phenology was calibrated first using the data from five years (2016–2020) and four locations, i.e., Rødby, Haderslev, Svenstrup, and Flakkebjerg. As observed, BBCH stages are similar to Zadok stages [53]; we renamed Zadok stages [54] output from APSIM as BBCH in the figures and tables presented here. Same dates as of BBCH stage observations in the field were extracted from simulation outputs and BBCH stages on those dates were used to compute RMSE, NSE, and r^2^ (Section 4.2). For the second step, biomass and N uptake, and for the third step, grain yield and grain N were calibrated. Model performance was computed (Section 4.2) after the third step of calibration, which is similar to full parameterization or a high level of calibration approach for best performance of crop models [55,56]. A comparison of the calibrated set of parameters in this study with the parameters of default winter wheat in APSIM 7.10 (cv. Batten_winter) and calibrated parameters in an earlier study in Denmark [25] is shown in the results. The cultivar with the calibrated set of parameters in this study will be named Dan_winter cultivar (signifies Danish winter wheat cultivar), Batten_winter as default cultivar, and the one modified parameters of Batten_winter in Vogeler et al. [25] as modified cultivar, hereafter. Most parameters that were calibrated in this study are cultivar-specific parameters, except SLAmax and initial_tpla, which are crop-specific parameters. The parameters SLAmax and initial_tpla were calibrated because only these two parameters were most effective and could reduce the overestimation of biomass during early growth stages. The calibrated parameters are presented in Table 5 with their default values. Due to the same maturity class, and only slight differences in grain yield and crude protein in dry matter of the cultivars, only one set of parameters was calibrated for the seven cultivars. This approach potentially makes the model applicable to a range of cultivars, while we acknowledge that accuracy may be compromised based on cultivar characteristics and growing conditions.

We would also like to mention that we tried to compute the values using observed data for potential_grain_filling_rate, potential_grain_growth_rate, and potential_grain_n_filling_rate using the default value for grains_per_gram_stem (i.e., 25). These computed values from the treatment of 300 kg N ha^−1^ (assumption of potential conditions) resulted in overestimation of grain yield and underestimation of grain N. Additionally, stem biomass tends to increase from the mid grain filling period towards maturity, which suggested sink limitation in the model (i.e., lower value for grains_per_gram_stem). Since we did not have recorded data for grain number per g stem weight, we went ahead with the trial-and-error approach to find the calibrated values of those four parameters for best calibrated balance of grain yield, grain N, and no sign of sink limitation.

### 4.7. Sensitivity Analysis

After the model calibration, we found that calibrated set of parameters tends to overestimate N uptake during early growth stages. Therefore, we performed a sensitivity analysis of parameters that regulate critical and maximum N concentration in the leaf and stem (i.e., n_conc_crit_leaf, n_conc_max_leaf, n_conc_crit_stem, and n_conc_max_stem), and N access and uptake from the soil (i.e., total_n_uptake_max and kno3) to find the reason for overestimation. The overestimation was primarily observed in early developmental stages; so, the sensitivity analysis was performed only on parameter values that regulate N concentration in the leaf and stem from emergence (BBCH 10) to the end of the juvenile stage (BBCH 32). The values were changed with a regular interval both higher and lower than the default values (Figures S1 and S2). For total_n_uptake_max and kno3, one but different values for each parameter is used for the whole crop duration. Similar to the above parameters, higher and lower values than the default ones, 0.6 and 0.02, respectively, for total_n_uptake_max and kno3 were changed in a regular interval for the sensitivity analysis (Figure 6). The sensitivity analysis was conducted for three years (2016–2018) under two contrasting applications of nitrogen 0 and 270 kg N ha^−1^ using the simulation setup of Flakkebjerg location to capture possible interaction with limited and non-limited N conditions.

### 4.8. Model Performance Determinants

Parameter values that simulated phenology, biomass, grain yield, and grain N closest to the observed data were identified by commonly used criteria, minimizing the root mean square error (RMSE, Equation (1)) [27]. Besides RMSE, the coefficient of determination (r^2^, Equation (2)) and Nash–Sutcliffe model efficiency coefficient (NSE, Equation (3)) were also computed to further evaluate the calibrated model for the overall relationship between simulation and measured data points. RMSE can be interpreted as the prediction error that is not resolved by the model, r^2^ indicates the goodness of fit, and NSE, the predictive power of the model. Ideally, RMSE should be close to zero, r^2^ and NSE close to 1, and NSE at least positive.

(1) $$RMSE=\sqrt{\frac{1}{n}\sum_{i=1}^{n}{\left(o_{i}-s_{i}\right)}^{2}}$$

(2) $$r^{2}=\frac{n\sum_{i=1}^{n}\left(s_{i}-o_{i}\right)-\sum_{i=1}^{n}s_{i}\sum_{i=1}^{n}o_{i}}{\sqrt{\left(n\sum_{i=1}^{n}s_{i}{}^{2}-\sum_{i=1}^{n}s_{i}\sum_{i=1}^{n}s_{i}\right)\;(n\sum_{i=1}^{n}o_{i}{}^{2}-\sum_{i=1}^{n}o_{i}\sum_{i=1}^{n}o_{i}})}$$

(3) $$NSE=1-\frac{\sum_{i=1}^{n}{\left(s_{i}-o_{i}\right)}^{2}}{\sum_{i=1}^{n}\left(o_{i}-O\right)}$$

## 5. Conclusions

Low availability of comprehensive data on winter wheat has limited detailed calibration and evaluation of crop models, particularly APSIM, which further limits their application for planning fertilizer management strategies in Northern Europe. Field trial data were obtained from two sources, (1) farmers’ fields and (2) research stations, to assess the performance of the APSIM-wheat model (hereafter model) to simulate the dynamics of winter wheat phenology, biomass, N uptake, grain yield, and grain N with various fertilizer applications at seven sites in Denmark. Calibrated parameters in the model simulated phenology from the early growth stages to maturity with a high accuracy both with calibration and evaluation data sets. Simulation accuracy for biomass accumulation and N uptake during the early growth stages (BBCH 28–49) was also satisfactory, with a high accuracy during target stages of fertilizer application in spring (BBCH 28–31) and booting (45–47). There were mixed responses to simulate biomass and N uptake for BBCH 32–39 with a tendency to overestimate the N uptake with a high fertilizer input. This was attributed to high inter-annual variability in the simulation outputs. The sensitivity analysis showed that high inter-annual variability may be linked with high sensitivity of parameters regulating the maximum total N uptake from the root zone and the individual soil layer in the model. Simulations for grain yield and grain N showed a higher accuracy with the low root mean square error than the simulations for biomass and N uptake during the early growth stages. Overall, the calibrated parameters captured the dynamics of phenology, biomass, N uptake, grain yield, and grain N satisfactorily and better than the default Batten_winter cultivar in APSIM and the recently modified Batten_winter in an earlier study. Considering the usual fertilizer application during the early growth stages in spring, the calibrated parameters in the APSIM-wheat model can be applied, which has rarely been attempted earlier, to develop fertilizer management strategies to optimize the N uptake and reduce N leaching to groundwater in Northern Europe. To further improve the simulation accuracy for the N uptake during the early growth stages, further research on the understanding of algorithm formalism for the interactions of climate–soil–plant (parameters regulating the N uptake from the soil) are suggested.

## Figures and Tables

**Table 1:** Performance of APSIM (7.10) winter wheat with current set of calibrated parameters (Dan_winter cultivar) under calibration and validation data sets in this study in comparison with default cultivar, and modified cultivar used in [25].

	Dan_Winter		Default Cultivar in APSIM (Batten_Winter)	Modified Cultivar Used in [25]
	Calibration	Validation		
Phenology				
r^2^	0.97	0.97	0.095	0.97
NSE	0.97	0.97	−0.77	0.95
* RMSE (BBCH)	4.15	3.98	30.3	5
Biomass at early growth stages (~28–47 BBCH)				
r^2^	0.65		0.59	0.57
NSE	0.47		0.4	0.39
RMSE (kg ha^−1^)	1510		1607	1615
N uptake at early growth stages (~28–60 BBCH)				
r^2^	0.66	0.64	0.62	0.63
NSE	0.57	0.24	0.46	0.58
RMSE (kg ha^−1^)	28	39	31	27
Grain yield at harvest				
r^2^	0.51	0.61	0.48	0.51
NSE	0.43	0.51	0.054	0.28
RMSE (kg ha^−1^)	1491	1296	1923	1674
Grain N: N uptake at harvest				
r^2^	0.6	0.688	−0.072	0.56
NSE	0.55	0.76	0.54	0.16
RMSE (kg ha^−1^)	32	25	50	44

* BBCH stages from the simulations were extracted based on the date of observations in the field.

**Table 2:** Inter- and intra-annual variability of simulated and observed biomass and N uptake. SD represents standard deviation. In parenthesis are percentages of the difference between SD of simulated and observed data over the observed data.

Category of Variability	Location	Year	BBCH * (Observed)	Applied Nitrogen (kg ha^−1^)		Biomass (kg ha^−1^)		N uptake (kg ha^−1^)	
						Observed	Simulation	Observed	Simulation
	Across locations	2018–2020	28–49	0–300	Mean	3169	4090	77	82
					SD	2062	2674	44	46
Inter-annual	Rødby	2018–2020	28–47	0–300	Mean	4298	3946	85	85
					SD	2124	2443	45	49
Inter-annual	Svenstrup	2019–2020	28–39	0–300	Mean	1516	2556	48	62
					SD	735	2218	24	42
Inter-annual	Haderselv	2019	31	300		845	406	37	16
		2020	31	300		1196	1680	31	55
					SD	248	901 (263%)	4	28 (550%)
Inter-annual	Svenstrup	2019	32	300		1166	2011	51	66
		2020	32	300		1453	2157	52	71
					SD	203	103 (49%)	0.7	3.5 (400%)
Intra-annual	Rødby	2020	31	200		3380	3392	88	94
			37	200		8067	7591	168	134
					SD	3315	2969 (10%)	57	28 (50%)
Intra-annual	Haderslev	2019	34	200		3639	3930	102	100
			37	200		5319	5180	128	116
					SD	1188	884 (26%)	18	11 (39%)

* Simulation data were extracted on the same dates when these BBCH stages were recorded in the fields. Simulations BBCH may necessarily be the same as observed BBCH.

**Table 3:** Measurement dates of the variables used for calibration and evaluation. DAS = days after sowing. The number of BBCH assessments that were performed during the crop cycle are mentioned in the parenthesis in phenology observation column.

Location	Year	Date of Measurement	DAS	Biomass	N Uptake	Grain Yield	Grain N	Phenology Observation (BBCH)
		Calibration data set						
Rødby	2018	23 May 2018	243	x	x			
		23 July 2018	304			x	x	
		23 May 2018	239	x	x			
		23 July 2018	300			x	x	24–90 (9 stages)
	2019	8 April 2019	203	x	x			
		23 April 2019	218	x	x			
		6 May 2019	231	x	x			
		27 July 2019	313			x	x	27–90 (9 stages)
	2020	31 March 2020	191	x	x			
		22 April 2020	213	x	x			
		11 May 2020	232	x	x			
		2 August 2020	315			x	x	23–90 (7 stages)
Haderslev	2018	2 August 2018	310			x	x	24–90 (7 stages)
	2019	3 April 2019	189	x	x			
		7 May 2019	223	x	x			
		14 May 2019	230	x	x			27–90 (6 stages)
		27 August 2019	335			x	x	
	2020	22 April 2020	209	x	x			
		13 May 2020	230	x	x			
		12 August 2020	321			x	x	31–90 (5 stages)
Svenstrup	2019	15 April 2019	195	x	x			
		29 April 2019	209	x	x			
		15 May 2019	225	x	x			
		30 August 2019	332			x	x	26–90 (8 stages)
	2020	14 April 2020	206	x	x			
		28 April 2020	220	x	x			
		27 May 2020	249	x	x			
		25 August 2020	339			x	x	31–90 (8 stages)
Flakkebjerg_T	2016	At final harvest				x	x	31–88 (11 stages)
	2017	At final harvest				x	x	30–85 (8 stages)
	2018	At final harvest				x	x	30–89 (10 stages)
Flakkebjerg_E	2016	At final harvest				x	x	32–88 (9 stages)
	2017	At final harvest				x	x	30–85 (8 stages)
	2018	At final harvest				x	x	30–89 (10 stages)
		Evaluation data set						
Brønderslev	2018	15 May 2018	252	x	x			
		29 May 2018	266	x	x			
		30 July 2018	328			x	x	26–90 (9 stages)
	2020	12 August 2020	320			x	x	32–90 (9 stages)
Horsens	2018	3 May 2018	236	x	x			
		16 May 2018	249	x	x			
		25 May 2018	258	x	x			
		23 July 2018	317			x	x	30–90 (9 stages)
Sæby	2018	15 May 2018	252	x	x			
		28 May 2018	265	x	x			
		3 August 2018	332			x	x	26–90 (9 stages)
Flakkebjerg_T and E	2019	1 May 2019	238	x				
		15 May 2019	252	x				
		27 May 2019	264	x				32–47 (3 stages)
		At final harvest				x	x	

**Table 4:** Soil characteristics of the locations used for calibration and evaluation of APSIM. Volumetric water content at permanent wilting point (water potential of −1.5 MPa), field capacity (water potential of −0.1 kPa), and saturation. APSIM was set up with five soil layers for up to 160 cm depth for each location. Depth of topsoil layers are provided and those of remaining individual layers increased between 15 cm and 80 cm with increasing depth of soil profile. Plant available water content (PAW) for entire soil profile.

Location	Depth (cm)	Bulk Densit (g/cc)	Wilting Point (mm/mm)	Field Capacity (mm/mm)	Saturation (mm/mm)	Organic Carbon (%)	pH	Sand (%)	Silt (%)	Clay (%)	$NO_{3}$^−^ (kg/ha)	$NH_{4}$^+^ (kg/ha)	PAW (mm)
Rødby	0–25	1.49	0.08	0.28	0.44	3.4	7.7	76	11	11	0.03	0.12	
	50–160	1.75	0.10	0.22	0.34	0.9	8.2	62	10	10	0.03	0.03	217
Haderselv	0–28	1.43	0.09	0.30	0.46	5.0	7.4	67	16	14	0.09	0.15	
	70–160	1.47	0.15	0.37	0.44	0.6	6.0	44	23	33	0.06	0.05	246
Svenstrup	0–22	1.50	0.08	0.30	0.43	4.5	6.2	77	12	8	0.08	0.15	
	50–160	1.90	0.07	0.22	0.28	0.5	6.4	79	8	14	0.09	0.05	187
Flakkebjerg	0–20	1.53	0.09	0.26	0.40	1.4	6.0	77	17	7	2.00	5.00	
	50–160	1.71	0.12	0.27	0.37	0.2	6.0	69	20	11	0.00	0.67	174
Bronderslev	0–28	1.31	0.08	0.41	0.45	3.7	7.5	57	30	7	0.08	0.15	
	60–160	1.54	0.02	0.29	0.38	0.4	5.7	78	19	3	0.07	0.05	325
Horsens	0–26	1.42	0.07	0.30	0.43	1.5	6.2	58	30	9	0.08	0.15	
	50–160	1.68	0.07	0.21	0.31	0.1	7.1	70	23	9	0.08	0.05	172
Sæby	0–30	1.30	0.08	0.35	0.44	2.4	6.8	51	36	9	0.09	0.15	
	60–160	1.81	0.09	0.26	0.30	0.1	5.6	58	28	14	0.08	0.05	223

**Table 5:** Parameters used for calibrating APSIM winter wheat phenology, biomass accumulation, grain yield, grain N, and N uptake.

Parameter	Unit	Parameter Description	Default Value	Calibrated Value
Phenology				
vern_sens	-	Sensitivity to vernalisaiton	1.5	4.65
photop_sens	-	Sensitivity to photoperiod	3	3.35
tt-end_of_juvenile	°Cd	Thermal time required from emergence to start of panicle/spikelet/floral initiation	400	450
tt_start_grain_fill	°Cd	Thermal time required from start of grain filling to end of grain filling	545	750
Biomass				
y_sla_max	mm^2^ g^−1^	Regulates specific leaf area	27,000, 22,000	24,500, 18,000
initial_tpla	mm^2^ plant^−1^	Intial plant leaf area after emergence	200	100
Grain Yield				
grains_per_gram_stem	grain/g stem weight	Regulates number of grains per gram of stem weight at the end of flowering (zadok stage 65)	25	37
max_grain_size	g	Regulates maximum weight of individual grain	0.041	0.045
potential_grain_filling_rate	g grain^−1^ day^−1^	Regulates potential daily grain filling rate from grain filling to maturity	0.002	0.0038
potential_grain_growth_rate	g grain^−1^ day^−1^	Regulates growth rate from flowering to start of grain filling	0.001	0.0006
Grain N				
potential_grain_n_filling_rate	g grain^−1^ day^−1^	Regulates potential daily N filling rate to grain from grain filling to maturity	0.000055	0.000035

## Data Availability

The article and Supplementary Materials contain all data.

## Supplementary Materials

The following supporting information can be downloaded at: https://www.mdpi.com/article/10.3390/plants12050986/s1, Table S1. Agronomic management in the field trials at Rødby, Haderslev, Svenstrup, Flakkebjerg from 2015 to 2020 used for calibrating the parameters of APSIM wheat model. Numbers in parenthesis are the amount of N applications in kg ha^−1^. At Flakkebjerg, crops were either sown on time (Flakkebjerg_T) or early (Flakkebjerg_E): Table S2. Agronomic management in the field trials conducted at Horsens, Sæby, and Bronderslev from 2018 to 2020 used for evaluating APSIM’s parameters. Numbers in parenthesis are the amount of N applications in kg ha^−1^: Figure S1. Sensitivity analysis of parameters regulating N concentration in leaf during early growth stages (from emergence to end of juvenile phase) for biomass (a), N uptake (b), grain yield (c), grain N (d), n_conc_crit_leaf, n_conc_max_leaf are parameters that regulate critical and maximum N concentration in leaf. The numbers in the parenthesis, for example in crit (0.030–0.030), indicate parameter n_conc_crit_leaf values from emergence (BBCH 10) to end of juvenile phase (BBCH 32). Simulation outputs are shown from BBCH 25 (mid of end of juvenile) to 90 (maturity): Figure S2. Sensitivity analysis of parameters regulating N concentration in stem during early growth stages (from emergence to end of juvenile phase) for biomass (a), N uptake (b), grain yield (c), grain N (d), n_conc_crit_stem, n_conc_max_stem are parameters that regulate critical and maximum N concentration in stem. The numbers in parenthesis, for example in crit (0.040–0.030), indicate parameter n_conc_crit_stem values from emergence (BBCH 10) to end of juvenile phase (BBCH 32). Simulation outputs are shown from BBCH 25 (mid of end of juvenile) to 90 (maturity).
