# Supplementary material for: Performance of APSIM to Simulate the Dynamics of Winter Wheat Growth, Phenology, and Nitrogen Uptake from Early Growth Stages to Maturity in Northern Europe
Source: Plants (Basel). 2023 Feb 21;12(5):986. doi: 10.3390/plants12050986 (PMC10005596; doi:10.3390/plants12050986)
Supplement: Supplementary file 1 [file plants-12-00986-s001.zip › plants-2137261-supplementary.pdf]

Table S1. Agronomic management in the field trials at Rødby, Haderslev, Svenstrup, Flakkebjerg from 2015 to 2020 used for calibrating the parameters of APSIM wheat model. Numbers in the parenthesis are the amount of N applications in kg ha^{-1}. At Flakkebjerg crops were either sown timely (Flakkebjerg_T) or early (Flakkebjerg_E)

Location	Rødby	Rødby	Rødby	Rødby	Haderslev	Haderslev	Haderslev	Svenstrup	Svenstrup	Flakkebjerg_T	Flakkebjerg_T	Flakkebjerg_T	Flakkebjerg_E	Flakkebjerg_E	Flakkebjerg_E
Sowing date	21/09/2017	25/09/2017	16/09/2018	21/09/2019	25/09/2017	25/09/2018	25/09/2019	1/10/2018	20/09/2019	29/09/2015	21/09/2016	25/09/2017	2/09/2015	2/09/2016	25/09/2017
Variety	Sheriff	Benchmark	Sheriff	KWS Extase	KWS Lili	Torp	Kvium	Torp	Blanding	Mariboss	Mariboss	Sheriff	Mariboss	Mariboss	Sheriff
Previous crop	Spring barly	Sugar beet	Spring barley	Spring barley	Winter rape	Winter rape	Winter wheat	Winter rape	Winter rape	Winter wheat	Winter wheat	Winter wheat	Winter wheat	Winter wheat	Winter wheat
	Year														
Fertilizer treatment	2018	2018	2019	2020	2018	2019	2020	2019	2020	2016	2017	2018	2016	2017	2018
1	No application	No application	No application	No application	No application	No application	No application	No application	No application	No application	No application	No application	No application	No application	No application
2	9-April (50)	10-April (50)	22-March (50)	9-March (50)	27-March (50)	12-March (50)	17-March (50)	26-March (50)	23-March (50)						
3	9-April (50)<br>26-April (50)	10-April (50)<br>26-April (50)	22-March (50)<br>8-April (50)	9-March (50)<br>30-March (50)	27-March (50)<br>24-April (50)	12-March (50)<br>4-April (50)	17-March (50)<br>23-April (50)	26-March (50)<br>17-April (50)	23-March (50)<br>18-April (50)	18-March (50)<br>20-April (51)	28-March (50)<br>25-April (51)	21-March (50)<br>29-April (51)	18-March (50)<br>20-April (51)	28-March (50)<br>25-April (51)	21-March (50)<br>29-April (51)
4	10-April (50)<br>26-April (100)	10-April (50)<br>26-April (100)	22-March (50)<br>8-April (100)	9-March (50)<br>30-March (100)	27-March (50)<br>24-April (100)	12-March (50)<br>4-April (100)	17-March (50)<br>23-April (100)	26-March (50)<br>17-April (100)	23-March (50)<br>18-April (100)	18-March (50)<br>20-April (102)	28-March (50)<br>25-April (102)	21-March (50)<br>29-April (102)	18-March (50)<br>20-April (102)	28-March (50)<br>25-April (102)	21-March (50)<br>29-April (102)
5	10-April (50)<br>26-April (150)	10-April (50)<br>26-April (150)	22-March (50)<br>8-April (100)<br>6-May (50)	9-March (50)<br>30-March (100)<br>6-May (50)	27-March (50)<br>24-April (150)	12-March (50)<br>4-April (100)<br>14-May (50)	17-March (50)<br>23-April (100)<br>14-May (50)	26-March (50)<br>17-April (100)<br>16-May (50)	23-March (50)<br>18-April (100)<br>25-May (50)	18-March (50)<br>20-April (152)	28-March (50)<br>25-April (152)	21-March (50)<br>29-April (152)	18-March (50)<br>20-April (152)	28-March (50)<br>25-April (152)	21-March (50)<br>29-April (152)
6	10-April (50)<br>26-April (200)	10-April (50)<br>26-April (200)	22-March (50)<br>8-April (150)<br>6-May (50)	9-March (50)<br>30-March (150)<br>6-May (50)	27-March (50)<br>24-April (200)	12-March (50)<br>4-April (150)<br>14-May (50)	17-March (50)<br>23-April (150)<br>14-May (50)	26-March (50)<br>17-April (150)<br>16-May (50)	23-March (50)<br>18-April (150)<br>25-May (50)	18-March (50)<br>20-April (202)	28-March (50)<br>25-April (202)	21-March (50)<br>29-April (202)	18-March (50)<br>20-April (202)	28-March (50)<br>25-April (202)	21-March (50)<br>29-April (202)
7	10-April (50)	10-April (50)	22-March (50)	9-March (50)	27-March (50)	12-March (50)	17-March (50)	26-March (50)	23-March (50)	18-March (50)	28-March (50)	21-March (50)	18-March (50)	28-March (50)	21-March (50)

	26-April (250)	26-April (250)	8-April (200)<br>6-May (50)	30-March (200)<br>6-May (50)	24-April (250)	4-April (200)<br>14-May (50)	23-April (200)	17-April (200)<br>16-May (50)	18-April (200)<br>25-May (50)	20-April (253)	25-April (253)	29-April (253)	20-April (253)	25-April (253)	29-April (253)
8	10-April (50)<br>26-April (100)<br>25-May (50)	10-April (50)<br>26-April (100)<br>25-May (50)	22-March (50)<br>8-April (150)	9-March (50)<br>30-March (100)	27-March (50)<br>24-April (100)<br>28-May (50)	12-March (50)<br>4-April (150)	17-March (50)<br>23-April (150)	26-March (50)<br>17-April (150)	23-March (50)<br>18-April (150)						
9			22-March (100)<br>8-April (200)	9-March (100)<br>30-March (200)		12-March (100)<br>4-April (200)	17-March (100)<br>23-April (200)	26-March (50)<br>17-April (200)	23-March (100)<br>18-April (200)						
10			22-March (50)<br>8-April (100)<br>6-May (100)	9-March (50)<br>30-March (100)<br>6-May (100)		12-March (50)<br>4-April (100)<br>14-May (100)	17-March (50)<br>23-April (100)<br>14-May (100)	26-March (50)<br>17-April (100)<br>16-May (100)	23-March (50)<br>18-April (100)<br>25-May (100)						
11			22-March (50)<br>8-April (100)<br>15-April (50)	9-March (50)<br>30-March (100)<br>15-April (50)		12-March (50)<br>4-April (100)<br>12-April (50)	17-March (50)<br>23-April (100)<br>March-May (50)	26-March (50)<br>17-April (100)<br>25-April (50)	23-March (50)<br>18-April (100)<br>April-May (50)						
12			22-March (50)<br>8-April (100)<br>23-April (50)	9-March (50)<br>30-March (100)<br>23-April (50)		12-March (50)<br>4-April (100)<br>19-April (50)	17-March (50)<br>23-April (100)<br>10-May (50)	26-March (50)<br>17-April (100)<br>02-May (50)	23-March (50)<br>18-April (100)<br>11-May (50)						
13			22-March (50)<br>8-April (100)<br>29-April (50)	9-March (50)<br>30-March (100)<br>30-April (50)		12-March (50)<br>4-April (100)<br>25-April (50)	17-March (50)<br>23-April (100)<br>19-May (50)	26-March (50)<br>17-April (100)<br>09-May (50)	23-March (50)<br>18-April (100)<br>18-May (50)						

Table S2. Agronomic management in the field trials conducted at Horsens, Sæby, and Bronderslev from 2018 to 2020 used for evaluating APSIM's parameters. Numbers in the parenthesis are the amount of N applications in kg ha^{-1}

Location	Bronderslev	Bronderslev	Horsens	Sæby	Flakkebjerg_T	Flakkebjerg_T	Flakkebjerg_E	Flakkebjerg_E
Sowing date	4/09/2017	26/09/2019	8/09/2017	4/09/2017	20/09/2018	19/09/2019	4/09/2018	4/09/2019
Variety	Benchmark	Informer	Torp	Torp	Mariboss	Mariboss	Mariboss	Mariboss
Previous crop	Winter wheat	Winter wheat	Winter wheat	Winter rape	Winter wheat	Winter wheat	Winter wheat	Winter wheat
Fertilizer treatment	Year							
	2018	2020	2018	2018	2019	2020	2019	2020
1	No application	No application	No application	No application	No application	No application	No application	No application
2	10-April (50)	16-March (50)	31-March (50)	10-April (50)				
3	10-April (50)<br>25-April (50)	16-March (50)<br>17-April (50)	31-March (50)<br>25-April (50)	10-April (50)<br>26-April (50)	01-April (50)<br>02-May (51)	25-March (100)<br>12-May (0)	01-April (50)<br>02-May (51)	25-March (100)<br>12-May (0)
4	10-April (50)<br>25-April (100)	16-March (50)<br>17-April (100)	31-March (50)<br>25-April (100)	10-April (50)<br>26-April (100)	01-April (50)<br>02-May (102)	25-March (100)<br>12-May (50)	01-April (50)<br>02-May (102)	25-March (100)<br>12-May (50)
5	10-April (50)<br>25-April (150)	16-March (50)<br>17-April (100)<br>19-May (50)	31-March (50)<br>25-April (150)	10-April (50)<br>26-April (150)	01-April (50)<br>02-May (152)	25-March (100)<br>12-May (100)	01-April (50)<br>02-May (152)	25-March (100)<br>12-May (100)
6	10-April (50)<br>25-April (200)	16-March (50)<br>17-April (150)<br>19-May (50)	31-March (50)<br>25-April (200)	10-April (50)<br>26-April (200)	01-April (50)<br>02-May (202)	25-March (100)<br>12-May (150)	01-April (50)<br>02-May (202)	25-March (100)<br>12-May (150)
7	10-April (50)<br>25-April (250)	16-March (50)<br>17-April (200)<br>19-May (50)	31-March (50)<br>25-April (250)	10-April (50)<br>26-April (250)	01-April (50)<br>02-May (253)	25-March (100)<br>12-May (200)	01-April (50)<br>02-May (253)	25-March (100)<br>12-May (200)
8	10-April (50)<br>25-April (100)<br>30-May (50)	16-March (50)<br>17-April (150)	31-March (50)<br>25-April (100)<br>24-May (50)	10-April (50)<br>26-April (100)<br>31-May (50)				
9	10-April (80)<br>7-May (80)<br>30-May (40)	16-March (50)<br>17-April (100)<br>19-May (100)	31-March (80)<br>7-May (80)<br>24-May (40)	10-April (80)<br>26-April (80)<br>31-May (40)				

10	10-April (50)<br>25-April (94)<br>30-May (91)	16-March (50)<br>17-April (100)<br>02-June (50)	10-April (50)<br>26-April (80)<br>31-May (46)
11	10-April (80)<br>7-May (93)<br>30-May (83)	16-March (50)<br>17-April (100)<br>02-June (100)	10-April (80)<br>26-April (61)<br>31-May (53)
12		16-March (100)<br>17-April (200)	
13		16-March (50)<br>4-May (100)<br>19-May (50)	

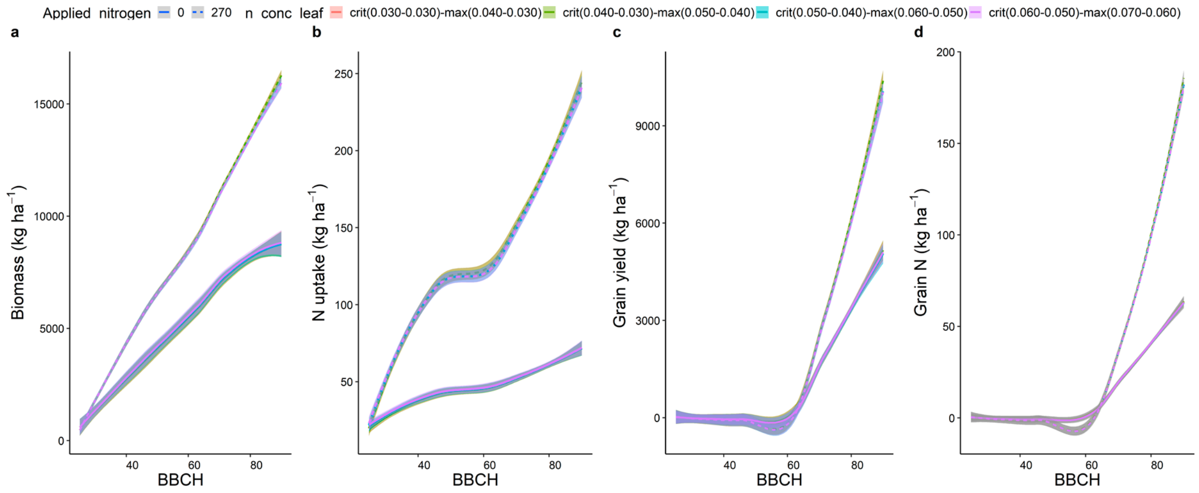

Figure S1. Sensitivity analysis of parameters regulating N concentration in leaf during early growth stages (from Emergence to End of Juvenile phase) for biomass (a), N uptake (b), grain yield (c), grain N (d). n_conc_crit_leaf, n_conc_max_leaf, are parameters that regulate critical and maximum N concentration in leaf. The numbers in the parenthesis, for example in crit(0.030-0.030), indicate parameter n_conc_crit_leaf values

from emergence (BBCH 10) to end of Juvenile phase phase (BBCH 32). Simulation outputs are shown from BBCH 25 (mid of end of juvenile) to 90 (maturity).

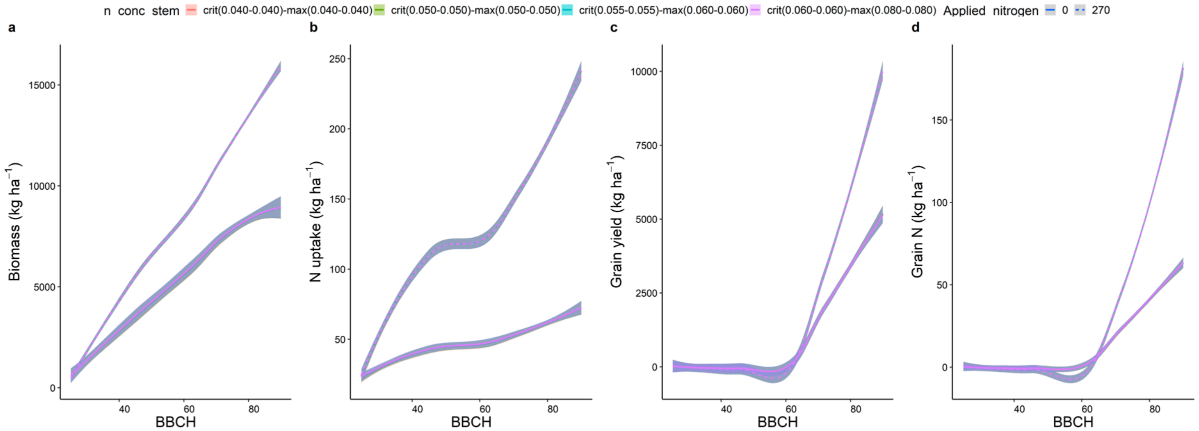

Figure S2. Sensitivity analysis of parameters regulating N concentration in stem during early growth stages (from Emergence to End of Juvenile phase) for biomass (a), N uptake (b), grain yield (c), grain N (d). n_conc_crit_stem, n_conc_max_stem, are parameters that regulate critical and maximum N concentration in stem. The numbers in the parenthesis, for example in crit(0.040-0.030), indicate parameter n_conc_crit_stem values from emergence (BBCH 10) to end of Juvenile phase phase (BBCH 32). Simulation outputs are shown from BBCH 25 (mid of end of juvenile) to 90 (maturity).
